# Improved Treatment Outcomes by Using Patient Specific Drug Combinations in Mammalian Target of Rapamycin Activated Advanced Metastatic Cancers

**DOI:** 10.3389/fphar.2021.631135

**Published:** 2021-04-16

**Authors:** Timothy Crook, Darshana Patil, Andrew Gaya, Nicholas Plowman, Sewanti Limaye, Anantbhushan Ranade, Amit Bhatt, Raymond Page, Dadasaheb Akolkar

**Affiliations:** ^1^ Broomfield Hospital, Chelmsford, United Kingdom; ^2^ Datar Cancer Genetics, Nasik, India; ^3^ HCA Healthcare United Kingdom, London, United Kingdom; ^4^ St Bartholomew’s Hospital, London, United Kingdom; ^5^ Kokilaben Dhirubhai Ambani Hospital, Mumbai, India; ^6^ Avinash Cancer Clinic, Pune, India; ^7^ Worcester Polytechnic Institute, Worcester, India

**Keywords:** encyclopedic tumor analysis, ETA, mTOR, PIK3CA, PI3K, Akt, mTOR inhibitor, rapalog

## Abstract

**Background:** Activation of the mTOR signaling pathway is ubiquitous in cancers and a favourable therapeutic target. However, presently approved mTOR inhibitor monotherapies have modest benefits in labeled indications while poor outcomes have been reported for mTOR inhibitor monotherapy when administered in a label-agnostic setting based on univariate molecular indications. The present study aimed to determine whether patient-specific combination regimens with mTOR inhibitors and other anticancer agents selected based on multi-analyte molecular and functional tumor interrogation (ETA: Encyclopedic Tumor Analysis) yields significant treatment response and survival benefits in advanced or refractory solid organ cancers.

**Methods:** We evaluated treatment outcomes in 49 patients diagnosed with unresectable or metastatic solid organ cancers, of whom 3 were therapy naïve and 46 were pre-treated in whom the cancer had progressed on 2 or more prior systemic lines. All patients received mTOR inhibitor in combination with other targeted, endocrine or cytotoxic agents as guided by ETA. Patients were followed-up to determine Objective Response Rate (ORR), Progression Free Survival (PFS) and Overall Survival (OS).

**Results:** The Objective Response Rate (ORR) was 57.1%, the disease Control rate (DCR) was 91.8%, median Progression Free Survival (mPFS) was 4.9 months and median Overall Survival (mOS) was 9.4 months. There were no Grade IV treatment related adverse events (AEs) or any treatment related deaths.

**Conclusion:** Patient-specific combination regimens with mTOR inhibition and other anti-neoplastic agents, when selected based on multi-analyte molecular and functional profiling of the tumor can yield meaningful outcomes in advanced or refractory solid organ cancers.

**Trial Registration:** Details of all trials are available at WHO-ICTRP: https://apps.who.int/trialsearch/. RESILIENT ID CTRI/2018/02/011808. ACTPRO ID CTRI/2018/05/014178. LIQUID IMPACT ID CTRI/2019/02/017548.

## Background

Mammalian Target of Rapamycin (mTOR) is a protein kinase which plays an important role in tumorigenesis by controlling protein synthesis, cell growth and proliferation and metastasis (Crespo et al., 2016). Since activation of the mTOR signaling pathway is ubiquitous in cancers, therapeutic inhibition of mTOR using analogs of Rapamycin (‘Rapalogs’) has been an attractive strategy for systemic management of cancer, albeit with modest benefits (Kwitkowski et al., 2010; Yao et al., 2011; Buti et al., 2016; Hua et al., 2019). Previous attempts to match alterations in mTOR pathway genes with label-agnostic mTOR blockade via monotherapy have reported inferior outcomes (Le Tourneau et al., 2015; Tsimberidou et al., 2019). The low efficacy of mTOR inhibitors has been attributed to the largely cytostatic rather than cytotoxic mechanisms of action (Meric-Bernstam and Gonzalez-Angulo, 2009), their limited inhibitory capacity as well as the activation of other resistance pathways (Faes et al., 2017). There is growing evidence that mTOR inhibitors in multi-drug combination regimens can overcome the largely cytostatic effect of mTOR inhibitor monotherapies thus leading to improved treatment outcomes especially in advanced cancers. Illustratively, the combination of Everolimus and Exemestane is superior to Everolimus alone in treatment of patients with non-steroidal aromatase-inhibitor refractory ER^+^/HER2^-^ metastatic breast cancer (Jerusalem et al., 2018). Likewise, the combination of Everolimus and lenvantinib has been approved for metastatic RCC (Leonetti et al., 2017) owing to higher efficacy over Everolimus monotherapy. Similarly, though Alpelisib monotherapy targeting mutant PIK3CA has shown limited efficacy (∼6% ORR) in solid organ cancers, the combination of Alpelisib and Fulvestrant has yielded higher response rates (∼26%) in ER+/HER2-metastatic breast cancers (Juric et al., 2018; André et al., 2019).

It is accepted that tandem therapeutic targeting of multiple signaling pathways can lead to improved outcomes in cancer (O’Reilly, 2002). The mTOR pathway cross-talks with multiple other signaling pathways such as MAKP/ERK (Mendoza et al., 2011; Liu et al., 2018), AR (Mulders, 2009) and VEGF (Crumbaker et al., 2017). Some crosstalk appears to be linked to resistance mechanisms, while a subset may present therapeutically relevant targets (Conciatori et al., 2018; Liu et al., 2018). Likewise, several other signaling pathways that are also known to be upregulated in cancers, offer additional opportunities for tandem therapeutic targeting (O’Reilly, 2002).

Although the potential benefits of Everolimus in combination with chemotherapy agents have been hypothesized in various cancers, the benefits of such regimens in a refractory setting has not yet been demonstrated. Further, selection of chemotherapy agents for such combination regimens have been largely derived from Randomized Clinical Trials (RCT) or Standard of Care (SoC) guidelines rather than via patient-specific evaluation of drug resistance or sensitivity in respective tumors. The benefits of the latter approach lie not only in identifying relevant drugs with higher antitumor activity (Jo et al., 2018) but also provide a repertoire of drugs that can be used in a label-agnostic setting.

The clinical utility of patient-specific multi-analyte tumor interrogation (called ETA for ‘Encyclopedic Tumor Analysis’) for identifying vulnerabilities in advanced refractory cancers (ARC) and their targeting with personalized *de novo* combination treatment regimens has been previously demonstrated (Nagarkar et al., 2019). Here, we report the efficacy of such personalized combination treatment regimens which achieve efficacious mTOR blockade as well as tandem targeting of other tumor vulnerabilities to yield meaningful outcomes in treatment of advanced refractory cancers.

## Methods

### Study Design

This manuscript reports data from a subset of patients from three prospective interventional phase II/III trials, including RESILIENT (CTRI/2018/02/011808), ACTPRO (CTRI/2018/05/014178) and LIQUID IMPACT (CTRI/2019/02/017548) who received mTOR inhibitor-based treatments. The primary outcome data for the RESILIENT Trial has already been published (Nagarkar et al., 2019). The outcome data for the other two trials will be reported separately. The present manuscript only reports findings in a subset of patients from these trials where the therapy profile is relevant to the theme of this submission. Details of all trials are available at WHO-ICTRP. All trials were approved by institutional review boards and ethics committees of sponsor as well as clinical trial site. All trials were conducted in accordance with all applicable ethical guidelines and the Declaration of Helsinki. The present manuscript also retrospectively reports data from a curated subset of patients who availed of Encyclopedic Tumor Analysis (ETA) as a commercial service offered by the study sponsor for personalized treatments; outcomes are reported only for those patients who received mTOR-inhibitor based treatments.

### Patients

Between Jan 2018 and Jun 2019, 37 patients with advanced solid organ cancers received treatments with mTOR inhibitors in combination with other systemic anticancer agents as part of various prospective interventional clinical trials conducted by the study sponsor. All study participants were previously counseled regarding study objectives, potential benefits and potential risks and provided signed written informed consent for participation in the trial and for publication of deidentified data. Between Jan 2018 and Dec 2018, 12 patients underwent ETA as a commercial service to inform precision systemic therapy options for advanced broadly refractory solid organ tumors and received treatments with mTOR inhibitors in combination with other systemic anticancer agents. Treatment outcomes were available in these patients and were hence considered for analysis. All patients consented for analysis and publication of deidentified data. Outcome data for these patients are reported.

### Encyclopedic Tumor Analysis

The process of ETA and generation of patient specific therapy recommendations have been described previously (Nagarkar et al., 2019) and is also provided as Supplementary Material. Briefly, ETA included molecular profiling of tumor tissue and blood samples by NGS, immunohistochemistry (IHC) on tumor tissue and *in vitro* chemoresponse profiling (CRP) of viable tumor tissue derived cells (TDCs) or Circulating Tumor Associated Cells (CTACs) from peripheral blood. Both cytotoxic anticancer agents as well as mTOR inhibitors were evaluated by CRP where viable TDCs/CTACs were treated *in vitro* with standardized concentrations of anti tumor agents and the proportion of cell death was measured. Next Generation Sequencing (NGS) analysis of tumor tissue DNA or peripheral blood circulating tumor DNA (ctDNA) using a targeted gene panel (452 or 411 genes) was performed to identify molecular alterations in the mTOR pathway genes that are known to be indicative for selection of mTOR inhibitor as well as molecular alterations to select appropriate targeted and endocrine agents. Finally, Immunohistochemistry (IHC) profiling of tumor tissue was used to determine expression of Estrogen Receptor (ER) and Androgen Receptor (AR) for selection of Endocrine agents. ETA findings were integrated to generate patient specific treatment recommendations which were shared with the treating oncologist.

### Treatments

All patients received individualized combination regimens with mTOR inhibitors and other targeted, endocrine or cytotoxic drugs which were informed by ETA findings. Among 39 patients where the combination regimen included ≥1 cytotoxic agents, the choice of single or multiple cytotoxic agents was based on reported safety information (AE profiles) of each individual cytotoxic agent (labeled toxicity), as well as phase I trial data of safety and toxicity of combinations. This safety information was used to anticipate/predict patient-wize expected AEs which was referred to while determining the appropriate starting dose as well as dose escalation in each patient. In all patients, the treatment agents were initially administered at lower (≤50%) doses, and were escalated based on an individualized dose escalation schedule. Other factors which guided patient-specific dosage and schedule included institutional guidelines and protocols as well as clinical assessment of the patients’ health. As per the treatment plan in the trials, patients were to be administered treatments until progression or death or dose limiting toxicity. Patients who showed durable response were maintained with suitable dose reduction as decided by the treating clinician. For non-trial patients, schedule and duration were determined by the treating clinician based on clinical assessment of patients’ health.

### Response Evaluation

Treatment response was assessed in all patients based on a baseline and follow-up radiological imaging (CT/PET-CT) as per RECIST 1.1 criteria (Eisenhauer et al., 2009) to determine Objective Response Rate (ORR), disease Control Rate (DCR), Progression Free Survival (PFS) and Overall Survival (OS). Patients in clinical trials underwent follow-up imaging scans after every two cycles of treatment or after every 8–12 weeks, whichever was longer. All radiological data were independently evaluated by an external expert radiologist who was blinded to the interpretation of the original radiologist. If the findings of the external expert radiologist concurred with the gross findings of the original radiologist, then the initially reported values were retained. In case of divergent findings, this was conveyed to the original radiologist for re-evaluation of the radiological scan data.

### Follow-Up

Patients were followed up until study termination or patient exclusion (death/loss to follow-up/withdrawal of consent) or until December 2020, to determine Progression Free Survival (PFS) as well as Overall Survival (OS). Post completion of study, patients were followed-up every 6 months for OS only. Patients who were not part of the clinical trials underwent follow-up imaging scans at intervals specified by the treating clinicians.

### Safety and Adverse Events

Treatment related AEs were prospectively obtained for trial patients during the trial. Treatment-related AEs for non-trial patients were obtained from patients’ clinical records which were provided by the treating clinician. All AEs were graded according to NCI-CTCAE v5 (NCI, NIH, DHHS, 2017) and reported. For patients in the clinical trials, as well as commercial patients AEs were managed by standard procedures according to institutional protocols.

## Results

### Study Cohort

The present manuscript reports outcomes in 49 patients (23 males, 26 females, median age 49 years) who received mTOR inhibitor-based treatment regimens informed by ETA (Table 1, Supplementary Dataset). This cohort includes prospective data of 37 cancer patients from clinical trials and retrospective data of 12 cancer patients who received ETA-guided treatment recommendation commercially from the sponsor. Among the 49 patients, three were therapy naïve (at presentation) whilst 46 had refractory cancers which had progressed following failure of multiple lines of prior systemic therapy.

**TABLE 1:** Patient Demographics. The Study population includes 49 patients who received ETA guided combination treatments with mTOR inhibitors. Patient data was aggregated from three clinical trials conducted by the study sponsor as well as patients who availed of ETA as a commercial service from the sponsor.

Parameter	mTOR_C	mTOR_CT	mTOR_T	Overall
**Gender**	14 + 6 = 20	9 + 10 = 19	0 + 10 = 10	23 + 26 = 49
Male + female = total				
**Age (years)**	45 (27–71)	54 (8–68)	50 (36–61)	49 (8–71)
Median (range)				
**Cancer type**				
Bile duct	1	-	-	1
Breast	1	6	8	15
Colorectum	1	1	-	2
Endometrium	-	1	-	1
Esophagus	1	1	-	2
Head and neck	4	3	-	7
Kidney	-	2	-	2
Liver	1	-	-	1
Lung	3	2	-	5
Melanoma	1	-	-	1
Ovary	1	1	2	4
Pancreas	2	-	-	2
Hair follicle	1	-	-	1
Prostate	-	1	-	1
Sarcoma	1	-	-	1
Testes	2	-	-	2
Yolk sac tumor	-	1	-	1

### Treatments

All patients were administered mTOR inhibitors as part of multi-drug regimens where the combinations included either ≥1 cytotoxic agent (*n* = 20), cytotoxic and other targeted/endocrine agents (*n* = 19), or ≥1 targeted or endocrine agents (*n* = 10). In this cohort, seven patients were AR+, three patients were ER+ and two patients were AR+, ER + by IHC. All anticancer drugs in the combination treatments were approved by the United States FDA for use as antineoplastic agents. Selection of all treatment agents (including mTOR inhibitors) was agnostic to the respective labeled indications. Patient-wize drugs and regimens are provided in Supplementary Dataset.

### Treatment Response

Among the 49 patients, 1 (2.0%) showed Complete Response (CR), 27 (55.1%) showed Partial Response (PR), 17 (34.7%) showed SD and 4 (8.1%) patients showed PD. The Objective Response Rate (ORR) in this sub-cohort was 57.1% and disease Control Rate (DCR) was 91.8%. Patient outcomes are summarized in Table 2. In three patients, failure of a prior line of Everolimus inhibitor monotherapy had led to a previous instance of PD; all three patients received ETA guided combination regimen in the present study and showed PR. Additional relevant or unique cases are discussed in the Supplementary Material. Patient-wize responses to treatment are provided in Supplementary Dataset. Findings of the original radiologist and the external expert radiologist were found to be concurrent with regards to determining gross treatment response (PR, SD and PD) in all cases and hence did not necessitate any re-evaluation.

**TABLE 2:** Gene variants indicative of mTOR activation. The table indicates the types of gene variants observed and the number of patients where the tumors harbored each type of gene variants. Indications in italicized text are probable indications.

Gene	Reported indications
PIK3CA	p.E545 K (8), p.H1047 R (3), p.E542 K (3), p.M1043I (1), p.E1034G (1), p.E726 K (1), p.N345 K (1), p.C420 R (1), p.Y1021C (1). CNV_ < 6 (3), c.*25T > C (1), p.Y343C (1), p.E110K (1), p.R693H (1)
PTEN	p.D24 N (1), p.D92G (1), c.4932 A > G (1), p.R130* (1), p.V166 A (1), p.R159 S (1), p.R47G (1), p.D326G (1), p.Y68C (1), CNV_1 (3). *c.6354G > A (1)*
STK11	CNV_1 (3), p.F354 L (1)
AKT1	p.E17 K (2)
AKT2	*CNV_3 (1)*
TSC2	p.F1510del (2), p.F1510del (1), p.R1743Q (1). *p.S174L (1)*
MTOR	p.M2327I (1), p.R1709H (1)
NF1	p.R1250Q (1), p.S340 F (1), p.Q1520* (1)
ARID1A	p.P1326_Q1327insQ (1), p.P1618 L (1), CNV_1 (1)

Among 20 patients who received mTOR inhibitors in combination with cytotoxic agents (mTOR_C), PR was observed in 10 patients (50%). Among 29 patients, the combination regimen included an additional targeted or endocrine agent (mTOR_T, mTOR_CT) for tandem blockade of other signaling pathways; 18 of these patients (62.1%) showed PR. Within these 29 patients, PR was observed in 6/10 (60%) patients where AR/ER was targeted in tandem with mTOR, 5/9 (55.5%) patients where the VEGF signaling pathway was a tandem target and in 6/9 (66.7%) patients where the EGFR/ERBB2 pathway was targeted along with mTOR.

### Progression Free Survival and Overall Survival

**FIGURE 1:**
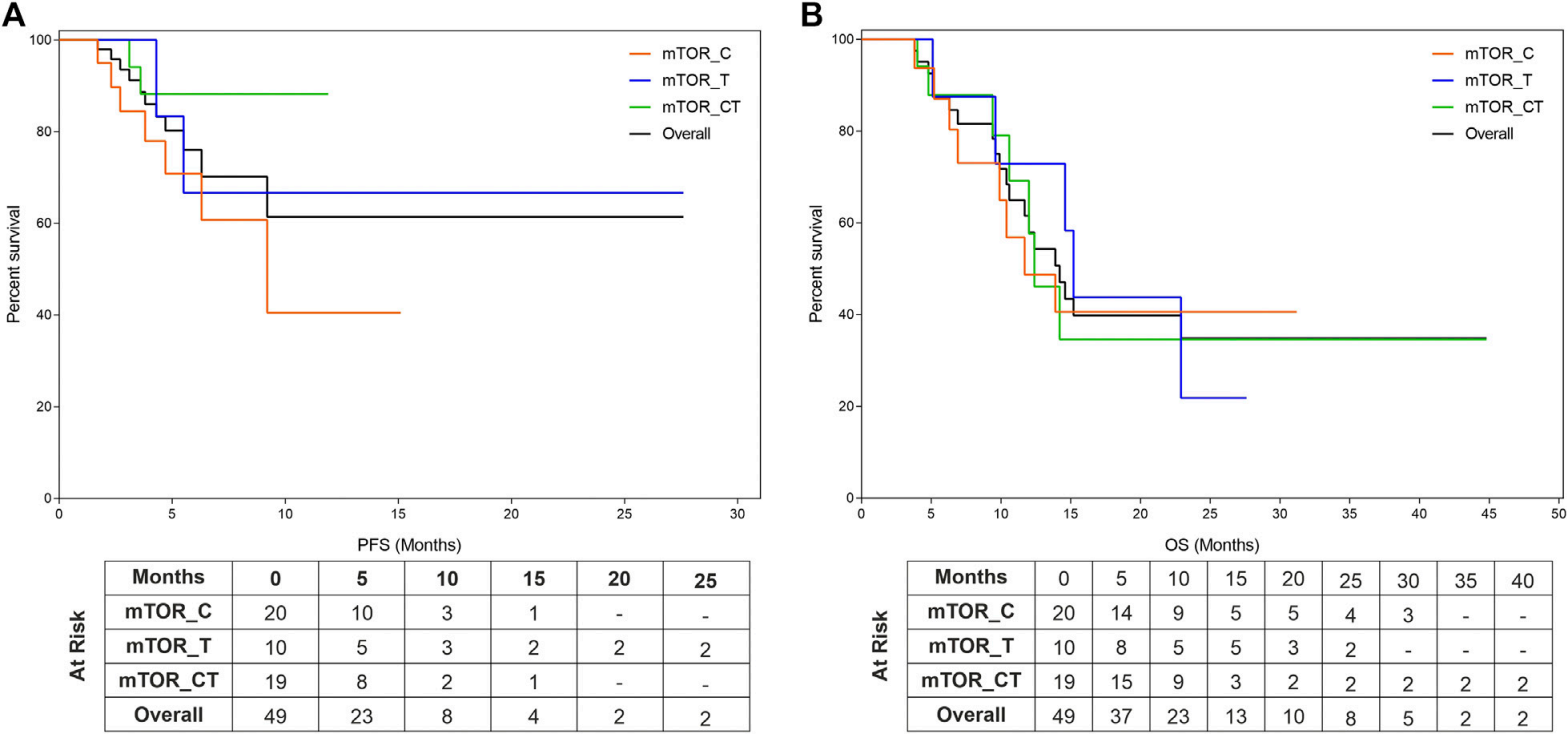
Kaplan Meier Plots of Progression Free Survival and Overall Survival. Progression Free Survival (PFS, **(A)**) and Overall Survival (OS, **(B)**) were evaluated for the entire cohort as well as subgroups which include mTOR inhibitors in combination with either Cytotoxic Agents (mTOR_C), other targeted agents (mTOR_T) or with both cytotoxic and targeted agents (mTOR_CT).

**FIGURE 2:**
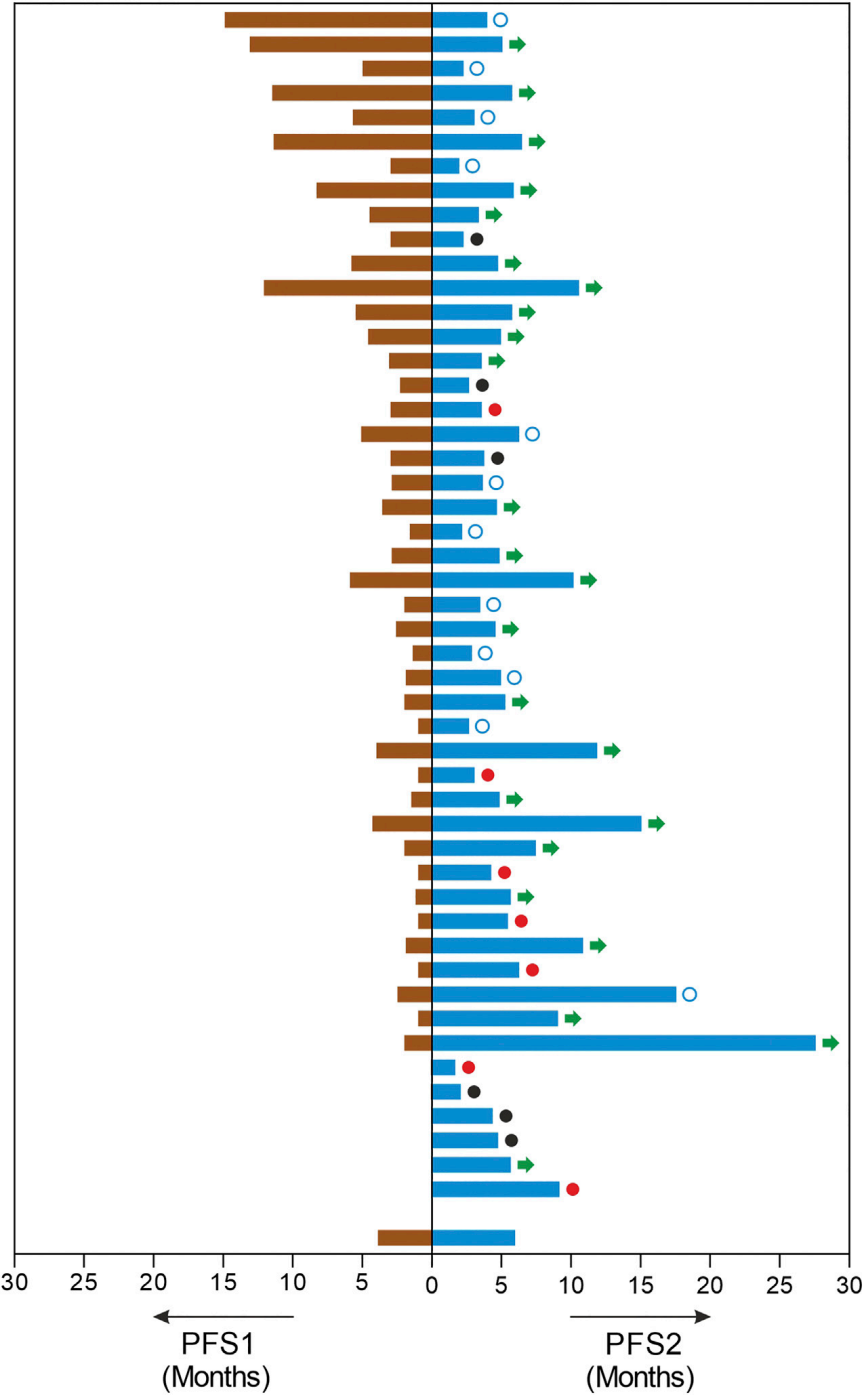
Improvements in Progression Free Survival. The image depicts each patients PFS in months on the last line of treatment (PFS1, left) and the PFS in months observed on ETA guided mTOR combination therapy regimen (PFS, right). In this cohort, three patients were therapy naïve and three patients had undergone prior surgery or radiation only. □: censored. ●: demise; ●: progression; →: ongoing PFS.

The study patients (*n* = 49) reported median PFS (mPFS) and median OS (mOS) of 4.9 months (95% CI: 3.6–6.2) and 9.4 months (95% CI: 6.6–12.2) respectively. The PFS rate and OS rate at 12 and 24 months were ∼60 and ∼35% respectively. The mPFS, PFS rates, mOS and OS rates in the various regimen subtypes are summarized in Table 3 along with the overall values. Kaplan Meier Plots of PFS and OS (overall as well as regimen subtypes are provided in Figure 1. In order to benchmark the benefits of ETA-guided therapy in the study cohort, we compared (Figure 2) the observed PFS of each patient on ETA-combination regimen (PFS2) against PFS on patient’s last failed line of therapy (PFS1). PFS2 was delimited due to demise in seven patients, due to disease progression in four patients, due to censoring in 14 patients (6 withdrew consent for further follow-up, eight defaulted). At the last follow-up, among the 24 patients who remained Progression Free, the ongoing Progression Free duration was reported as interim PFS. Based on these cut-offs, the PFS2:PFS1 ratio was ≥2.5 in 20 patients, between 1.3–2.5 in 10 patients, and ∼1 in six patients. The median PFS1 was 2.8 months, median PFS2 was 4.9 months and the overall PFS2:PFS1 ratio was 1.8, indicating that the median improvement was a significant extension of PFS over the last treatment. Patient-wize PFS and OS are provided in Supplementary Dataset.

**TABLE 3:** Treatment Outcomes. Progression Free Survival and Overall Survival Data are censored at the last follow-up.

	**mTOR_C**	**mTOR_CT**	**mTOR_T**	**Overall**
**Response**				
CR / PR	10	11	7	28
SD	8	6	3	17
PD	2	2	-	4
**Response Rates (%)**				
ORR	50.0	57.9	70.0	57.1
DCR	90.0	89.5	100.0	91.8
**Survival (months)**				
mPFS*	5.2 (3.8–6.6)	4.9 (3.8–6.0)	4.9 (2.0–10.0)	4.9 (3.6–6.2)
mOS*	7.2 (2.7–11.7)	9.4 (4.6–14.2)	12.1 (6.7–17.5)	9.4 (6.6–12.2)
**Survival Rates (%)**				
12-month PFS	40.0	85.0	65.0	60.0
12-month OS	55.0	70.0	75.0	55.0
24-month OS	40.0	35.0	20.0	35.0

CR, Complete Response; PR, Partial Response, SD, Stable Disease; PD, Progressive Disease; ORR, Objective Response Rate; DCR, Disease Control Rate; PFS, Progression Free Survival; OS, Overall Survival; mPFS, median PFS, mOS: median OS. *values within parentheses indicate 95% Confidence Interval.

### Molecular Alterations in the Mammalian Target of Rapamycin Pathway

**FIGURE 3:**
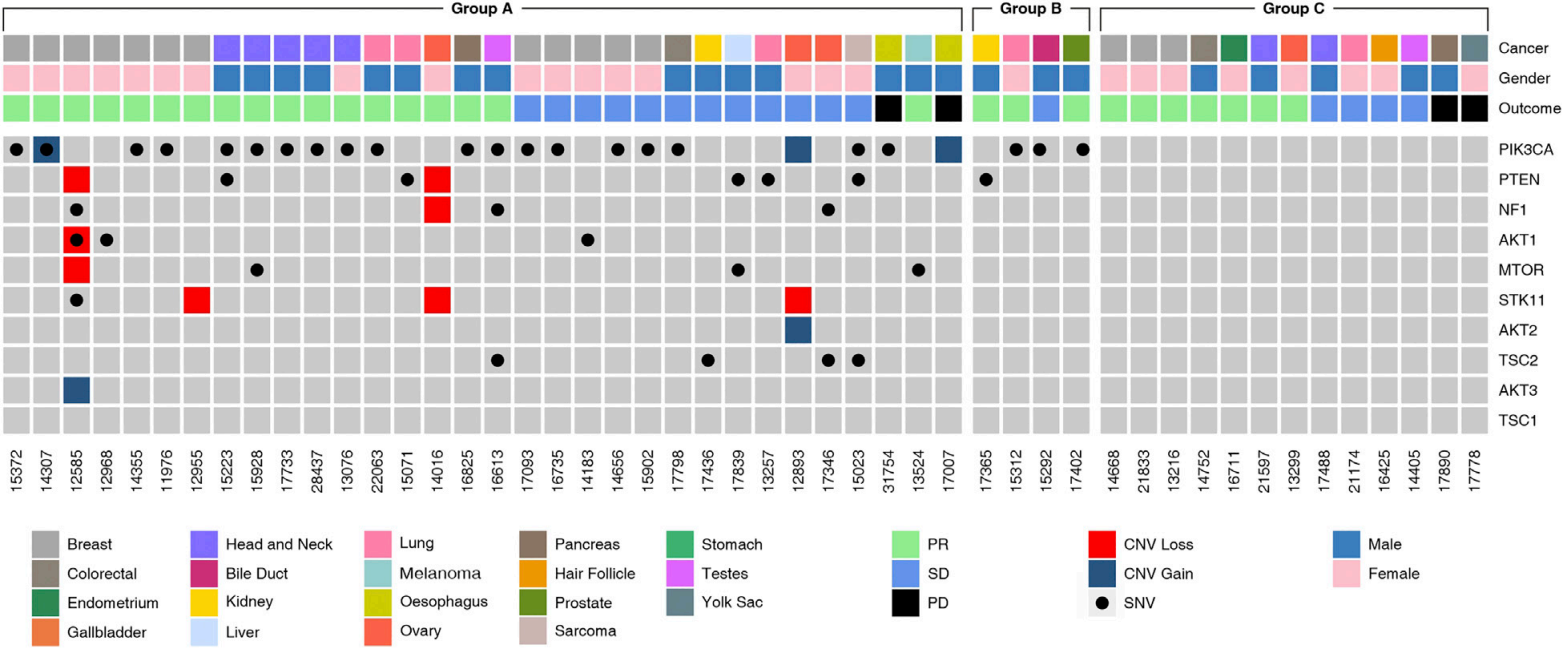
Molecular Landscape of Study Cohort**.** Molecular alterations observed in genes in the mTOR pathway genes as determined by Next Generation Sequencing (NGS) analysis of tumor tissue DNA or cell free tumor DNA (ctDNA) are depicted. Five-digit numbers at the bottom of each column indicate individual patients in the study cohort. Cancer types (topmost row) and gender (second row from top) are color coded. SNV and CNV (gain or loss) are color coded. The Study Cohort consisted of 49 patients divided into various sub-groups:Group A (*n* = 31, known ± probable mTOR activating variants), Group B (*n* = 5, probable mTOR activating variants) and Group C (*n* = 13, no known mTOR activating variants).

The molecular landscape of mTOR pathway associated genes in the study cohort as determined by NGS is depicted in Figure 3. Variations in PIK3CA and PTEN were most common among all genes related to the mTOR pathway. In 27 patients, the tumor harbored gene variants which were previously reported to be indicative for mTOR activation. In five patients, the tumors harbored gene variants which were probably indicative for mTOR activation, in addition to known targetable variants. In four patients, the tumor harbored no known targetable variants and only probably indicative variants. The phenotypic consequence of the variations appeared to be aligned with the activity profile of other known variants, and hence deemed as probable indications for mTOR inhibitor selection. Finally, in 13 patients there were no known gene variants indicative of mTOR activation.

### Adverse Events

There were no grade IV treatment related Adverse Events (AEs) or any treatment related deaths. Grade III treatment related AEs were seen in 34 patients. The most common grade III treatment related AEs were Fatigue (27%), Anorexia (11%) and Oral Mucositis (8%) which were managed by administration of standard treatment modalities Hyperglycemia which has been previously reported in mTOR regimens was observed in one patient. Patient-wize AEs are provided in Supplementary Dataset.

## Discussion

The study outcomes support the hypotheses of the study that ETA-guided combination regimens of mTOR inhibitors with other anti-neoplastic agents can achieve meaningful response in advanced refractory cancers especially when such combinations include other targeted/endocrine agents for tandem blockade of other tumor-associated signaling pathways. While PFS rates were higher in combination regimens that included another targeted agent (mTOR_T, mTOR_CT), the OS rates were similar across all therapy regimen subtypes indicating that mTOR inhibitors in combination regimens offer OS benefits while tandem targeting of additional tumor pathways yields PFS benefits as well apart from to OS benefits. It is generally accepted that subsequent lines of anticancer treatments are associated with decreasing probability of success. However. among patients who received ETA-guided combination regimens, there was an almost doubling of the PFS (PFS2:PFS1 ratio) indicating significant therapeutic benefit to patients. The authors acknowledge that the instances of censored PFS may underrepresent the actual extent of benefit. However, the recorded data indicate a significant median advantage despite censored observations; since therapy was ongoing in several patients, eventual improvements to these ratios are anticipated. We hence conclude that ETA guided combination regimens can provide significant PFS improvements even in heavily pre-treated populations. The response and survival benefits indicate the ability of ETA guided combination treatments to exploit known targetable vulnerabilities as well as to overcome known resistance variants. The present outcomes are remarkable in context of the PFS and ORR reported for the mTOR arm in the SHIVA trial (Le Tourneau et al., 2015), as well as outcomes in the NCI Match arms where modest benefits were observed such as 23% ORR for Capivasertib (Kalinsky et al., 2018), 0% ORR and 27% 6-months PFS rate for Taselisib (Krop et al., 2018) and 4% ORR and 1.8 months median PFS for GSK2636771 (Janku et al., 2018).

Presently, apart from Alpelisib, selection of other mTOR inhibitors is not based on molecular indications. Prior attempts to match variations in mTOR pathway genes with label-agnostic mTOR blockade (such as the trials mentioned above) have reported largely discouraging outcomes. Several variations are associated with mTOR activation such as alterations in the AKT (1/2/3), PIK3CA and PTEN genes besides the mTOR gene itself (Grabiner et al., 2014). Among the 32 patients with known and probable mTOR activation, the most common gene variants associated with the mTOR pathway were SNVs in PIK3CA (*n* = 24, 48.9%), loss of PTEN gene function via SNV or CNA (*n* = 9, 18%). Deleterious SNV/CNA in multiple mTOR pathway genes were also observed in some patients (*n* = 10, 20.4%). The present study does not aim to establish the predictive efficacy of these mTOR pathway variations for mTOR inhibitor selection or treatment response; the profile of (detected and undetectable) molecular variants in the known mTOR pathway genes suggests the role of additional hitherto unidentified genes and gene-variants linked to resistance or response toward mTOR inhibitors. Molecular alterations (SNV and CNV) of unknown significance in mTOR pathway genes were detected in 9 cases. These variations are speculated to be probable indications, which may be confirmed based on future insight into the phenotypic consequence (gain/loss of function) of these variations.

Prior attempts to identify potentially synergistic and safe anticancer drug combinations (Wang and Sorger, 2017; Sidorov et al., 2019; Zagidullin et al., 2019) as well as to predict drug efficacy via *in vitro* CRP of cell lines or primary tumor cellsy (Hurvitz et al., 2015; Mercatali et al., 2016; Kuo et al., 2019) reflect a consensus in favor of personalized combination regimens based on molecular and functional evidence. However, these prior reports do not have any correlation with clinical outcome data. In this regard, ours is the first report that provides clinical evidence demonstrating the utility and efficacy of a comprehensive, integrational multi-analyte-based approach (ETA) for informing personalized combination regimens. In ETA, targeted agents were selected based on NGS findings (SNV, CNA and Differential Gene Expression, DGE), endocrine agents were selected on the basis of hormone receptor (ER/AR) expression as determined by immunohistochemistry (IHC) on tumor tissue. mTOR inhibitors and cytotoxic agents were selected on the basis of *in vitro* CRP of viable TDCs or CTACs. It is pertinent to note that the observed drug efficacies by *in vitro* CRP is a summation of all known and latent resistance mechanisms including tumor-specific pathways as well as transiently dysregulated pathways. Since mTOR activation is associated with resistance to chemotherapy agents, *in vitro* CRP identified efficacious cytotoxic anticancer agents to which the tumor had not acquired resistance despite mTOR activation. Since the primary indication for mTOR inhibitor selection was *in vitro* CRP rather than molecular variations, ETA thus identified several patients (*n* = 13) with *in vitro* and largely *in vivo* response to mTOR inhibition, but where the tumor harbored no known alterations indicative of mTOR activation.

Having established the utility of ETA for selection of efficacious combination treatments with mTOR inhibitors and other antineoplastic agents, it is pertinent to review the safety of such *de novo* drug combinations. The safety profile of multi-drug anticancer regimens especially those with combinations of targeted and cytotoxic agents has been discussed at length in prior meta analyses (Liu et al., 2016; Nikanjam et al., 2016; Nikanjam et al., 2017). These studies observe that it is been possible to safely administer *de novo* (targeted and cytotoxic) drug combinations in most patients with manageable profiles of adverse events (AEs). It is generally agreed that although the actual profile of Adverse Events (AEs) in any given patient cannot be accurately predicted, the commonly occurring AEs associated with each drug or combinations can be anticipated. The profile of AEs shows that even though this heavily pretreated cohort was at an inherently higher risk of AEs due to cumulative toxicities from prior treatments, ETA guided therapies were generally well tolerated with a manageable toxicity profile (Li et al., 2015; Wilks, 2015).

The present study was largely based on a heavily pretreated cohort with minimal representation of therapy naïve patients. Hence, we are unable to demonstrate the benefits of ETA guided combination regimens as initial line therapy in treatment naïve patients at presentation.

To conclude, the present study is the first to demonstrate that ETA-guided combination regimens with mTOR inhibitor and other anticancer agents yield superior response rates and survival benefits as compared to mTOR inhibitor as monotherapy or in physician’s choice of combination regimens, in a (mostly) heavily pretreated cohort of patients with acceptable toxicity profile.

## Conclusion

We demonstrate that patient-specific combination regimens which achieve mTOR blockade and tandem targeting of other tumor vulnerabilities not only lead to favourable outcomes in advanced refractory cancers but also had manageable toxicity profiles. While prior attempts to expand the scope of mTOR inhibitor monotherapy in an organ agnostic setting based on univariate molecular profiling have been largely successful, we show that personalized combination regimens based on multi-analyte tumor profiling can yield significant and meaningful treatment benefits in various solid organ cancers. This is a viable pan-cancer treatment strategy since it overcomes the limited efficacy of mTOR inhibitors as well as the drug-resistance associated with activation of mTOR.

## Data Availability

The original contributions presented in the study are included in the article/Supplementary Material, further inquiries can be directed to the corresponding author.

## Glossary

mTOR: mammalian target of rapamycin
ETA: encyclopedic tumor analysis
ORR: objective response rate
DCR: disease control rate
mPFS,: median progression free survival
mOS: median overall survival
ER: estrogen receptor
HER2: human epidermal growth factor receptor 2
MAKP: mitogen activated protein kinase
ERK: extracellular signal-regulated kinase
AR: androgen receptor
VEGF: vascular endothelial growth factor
RCT: randomized clinical trial
SoC: standard of care
ARC: advanced refractory cancers
IHC: immunohistochemistry
USFDA: united states food and drug administration
CR: complete response
PR: partial response
SD: stable disease
PD: progressive disease (disease progression)
EGFR: epidermal growth factor receptor
ERBB2: synonym of HER2
PFS: progression free survival
OS: overall survival
AE: adverse event
CRC: colorectal cancer
ctDNA: circulating (cell-free) tumor DNA
SNV: single nucleotide variations
CNV: copy number variations
CRP: chemoresponse profiling
NGS: next generation sequencing
DGE: differential gene expression
TDCs: tumor tissue derived cells
C-TACs: circulating tumor associated cells
RECIST: response evaluation criteria in solid tumors
CTCAE: common terminology criteria for adverse events
